# Secretion of a low and high molecular weight β-glycosidase by *Yarrowia lipolytica*

**DOI:** 10.1186/s12934-020-01358-5

**Published:** 2020-05-11

**Authors:** Paul Swietalski, Frank Hetzel, Ines Seitl, Lutz Fischer

**Affiliations:** Department of Biotechnology and Enzyme Science, Institute of Food Science and Biotechnology, University of Hohenheim, Garbenstr. 25, 70599 Stuttgart, Germany

**Keywords:** *Yarrowia lipolytica*, PO1f, CRISPR Cas9, Protein secretion, Recombinant enzyme production, Yeast expression system, Unconventional protein secretion, CelB, M1

## Abstract

**Background:**

The secretory production of recombinant proteins in yeast simplifies isolation and purification but also faces possible complications due to the complexity of the secretory pathway. Therefore, correct folding, maturation and intracellular transport of the recombinant proteins are important processing steps with a higher effort needed for complex and large proteins. The aim of this study was to elucidate the secretion potential of *Yarrowia lipolytica* for low and high molecular weight β-glycosidases in a comparative cultivation approach.

**Results:**

A low sized β-glucosidase from *Pyrococcus furiosus* (CelB; 55 kDa) and a large sized β-galactosidase isolated from the metagenome (M1; 120 kDa) were integrated into the acid extracellular protease locus using the CRISPR–Cas9 system to investigate the size dependent secretion of heterologous proteins in *Y. lipolytica* PO1f. The recombinant strains were cultivated in the bioreactor for 78 h and the extra- and intracellular enzyme activities were determined. The secretion of CelB resulted in an extracellular volumetric activity of 187.5 µkat_oNPGal_/L_medium_, while a volumetric activity of 2.98 µkat_oNPGal_/L_medium_ was measured during the M1 production. However, when the amount of functional intra- and extracellular enzyme was investigated, the high molecular weight M1 (85%) was secreted more efficiently than CelB (27%). Real-time PCR experiments showed a linear correlation between the transcript level and extracellular activity for CelB, while a disproportional high mRNA level was observed regarding M1. Interestingly, mass spectrometry data revealed the unexpected secretion of two endogenous intracellular glycolytic enzymes, which is reported for the first time for *Y. lipolytica*.

**Conclusion:**

The results of this study provide deeper insights into the secretion potential of *Y. lipolytica*. A secretion limitation for the low-size CelB was observed, while the large size M1 enzyme was produced in lower amounts but was secreted efficiently. It was shown for the first time that *Y. lipolytica* is a promising host for the secretion of heterologous high molecular weight proteins (> 100 kDa), although the total secreted amount has to be increased further.

## Background

Recombinant protein production constitutes one of the key branches of current industrial biotechnology due to benefits in yield and costs [1, 2]. Bacteria, yeasts and molds are the hosts most often used in the field of enzyme production, producing more than 80% of industrial enzymes [3]. Thereby, the heterologous proteins can be either produced in the cytoplasm of the host or secreted outside the cell into the culture medium. Cytoplasmic production often leads to very high expression levels but comprises additional steps in downstream processing, such as cell disruption and lysate purification. To circumvent this, recombinant proteins should be secreted using a suitable signal sequence for the protein and an appropriate host [1]. However, secretory pathways are highly complex systems requiring many assisting proteins for maturation, folding and secretion [2]. The widely used yeast S*accharomyces cerevisiae* has a limited potential for the secretion of recombinant proteins, since these proteins were often retained inside the cell although a signal sequence for secretion was present [4, 5]. Therefore, other promising yeasts for the secretory production of recombinant proteins, such as *Komagataella phaffii* (formerly: *Pichia pastoris*), *Hansenula polymorpha, Kluyveromyces lactis* and the oleaginous yeast *Yarrowia lipolytica*, were investigated. In particular, the yeast *Y. lipolytica* has a naturally high capacity for the secretion of lipases, esterases, RNases and peptidases [6–10]. The alkaline extracellular protease was secreted up to 1–2 g/L under optimized conditions [11]. Not only the amount, but also the size of some described proteins which were natively secreted by *Y. lipolytica,* like the β-glucosidase Bgl2 (93 kDa) [12] makes this yeast an interesting universal production host, especially for large proteins. Consequently, more than 130 heterologous proteins from more than 80 species have been functionally produced in *Y. lipolytica* [13]. Proteins are secreted in *Y. lipolytica* predominantly by the co-translational pathway [14]. Another feature of *Y. lipolytica* is the total number of its natural proteins carrying a signal sequence (299), which is twofold higher than *S. cerevisiae* (156) [15]. A comparative study where six extracellular proteins were recombinantly produced in five different yeast species reported the extraordinary secretion potential of *Y. lipolytica* [16]. The functional secretion of lipase I from *Thermomyces lanuginosus*, for example, was tenfold higher in *Y. lipolytica* compared to *S. cerevisiae* and even 44-fold higher compared to cellulase II from *Humicola insolens* [16]. However, this study only investigated the functional secretion of small proteins ranging from 25 to 38 kDa. The recombinant secretion of higher molecular weight enzymes, such as the endoglucanase I from *Trichoderma reesei* (50 kDa), showed a 150-fold higher enzyme yield in *Y. lipolytica* compared to *S. cerevisiae* [17, 18]. Another study investigated the different secretion levels of endoglucanase II (50 kDa) and cellobiohydrolase II (65 kDa) from *T.* *reesei* in *K. phaffii* and *Y.* *lipolytica*, respectively [19]. Whereas the amount of the smaller endoglucanase II was quite similar for both hosts (15 mg/L in *Y.* *lipolytica* and 20 mg/L in *K. phaffii*) the yield for the larger cellobiohydrolase II, was almost doubled in *Y. lipolytica* (50 versus 27 mg/L) [19]. The largest proteins which have been secreted heterologously by *Y. lipolytica* to date are a glucoamylase from *Arxula adeninivorans* and the amino peptidase II from *Aspergillus oryzae*, both with molecular weights of ~ 90 kDa [20–22]. In our study, we compared the secretion of a low and high molecular weight β-glycosidase, which were both transcriptionally fused to the XPR2 signal peptide from the alkaline extracellular protease gene [23] by determining the intra- and extracellular activities during bioreactor cultivation and investigated the transcript level of the recombinant genes by real-time PCR.

The two glycosidases are promising candidates for applications in the dairy industry, such as lactose hydrolysis [24, 25] or lactulose production [26]. The β-glucosidase from the hyperthermophilic archaeon *Pyrococcus furiosus* (CelB, EC: 3.2.1.21) with a size of 55 kDa (monomer size; active as homotetramer) was selected as the low molecular weight glycosidase. This enzyme belongs to the family 1 glycosidases, having β-glucosidase (100%) and β-galactosidase activity (60%) [27]. CelB was already heterologously produced by *Escherichia coli* [28–30], *Lactobacillus casei* and *Lactobacillus plantarum* [31] inside the cell, but also secretorily by *K. phaffii* [32]. The highest protein level was achieved with *K.* *phaffii,* where apparently 740 mg/L of total protein was obtained in the supernatant [32]. The β-galactosidase M1 (EC: 3.2.1.23) was the other glycosidase selected as a high molecular weight example, 120 kDa in size. This β-galactosidase was isolated from a soil metagenome and shows favorable kinetic properties for lactose hydrolysis in milk at low temperatures [24]. Up to now, M1 has only been produced intracellularly in *E. coli* [24]. Therefore, an alternative production host, such as the food grade *Y. lipolytica*, would be essential for usage in the dairy industry.

## Results

### Integration of glycosidase genes into the acid extracellular peptidase locus of PO1f

The genes celB from *Pyrococcus furiosus* (KF420204; 1419 bp) and M1 from the metagenome [24] (KM651891; 3120 bp) were integrated within an identical expression cassette into the AXP locus of *Y. lipolytica* PO1f to compare the size-dependent mRNA expression and protein secretion level. Both genes were fused to the same XPR2 signal peptide [23]. Integration by homologous recombination (HR) was supported by the CRISPR Cas9 system using the method of markerless gene integration, according to Schwartz et al. [33]. Firstly, integration at the multifunctional enzyme locus 1 (MFE1) in PO1f was attempted, but without success (data not shown). Integration of both recombinant genes failed despite several trials. Since Schwartz and coworkers tested different integration sites [33], the flanking regions for HR were exchanged to sequences homolog to the AXP site, resulting in the vector pHR_AXP, and used a respective sgRNA for targeted double-strand break by the Cas9 endonuclease (Additional file 1: Table S1). After transformation of the CRISPR and HR plasmids (Additional file 1: Table S1) into PO1f, positive transformants were selected by the auxotrophic marker genes *ura3* and *leu2*. Genomic PCR screening was carried out to analyze the integration rate of both constructs. Significant differences in integration rates were obtained for CelB and M1. A high integration rate was achieved for the CelB expression cassette, where 80% of the colonies showed a positive integration (expression cassette detected/not detected). On the other hand, M1 was integrated only in 30% of the clones. Hence, we observed an influence of insert size on DNA integration efficiency, although the locus and the flanking regions were identical. This suggests a correlation between the length of the DNA inserted and the number of positive recombination events in *Y. lipolytica,* as was observed for other organisms [34].

### Production of heterologous *β*-glycosidases during bioreactor cultivation of PO1f strains

**Fig. 1:**
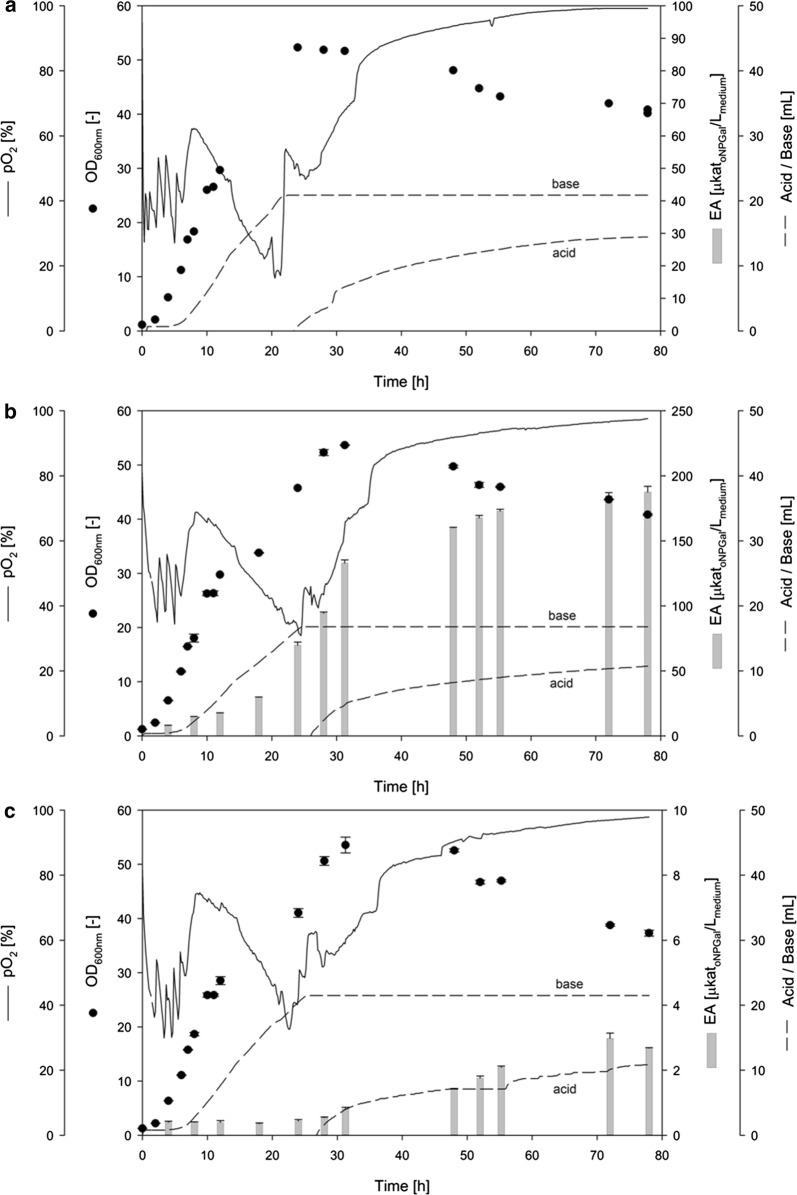
Bioreactor cultivation of *Y. lipolytica* PO1f (**a**), PO1f-CelB (**b**) and PO1f M1 (**c**). The cultivations were carried out in YPD medium at pH 6.5 and 0.5 vvm air for 78 h. OD_600_ [−] and extracellular β-galactosidase activity [µkat_oNPGal_/L_medium_] is indicated as black circles and grey bars, respectively

The two recombinant *Y. lipolytica* strains PO1f-CelB and PO1f-M1 and the unmodified PO1f strain (reference) were cultivated in bioreactors under equal conditions in YPD medium for 78 h (Fig. 1). All three strains showed generally similar growth behaviors. The exponential growth phases finished at 24 to 30 h with specific growth rates *µ* varying from 0.3 to 0.5 h^−1^. All three strains reached a similar maximum OD_600_ of ~ 53 (corresponding to a dry biomass (DBM) of ~ 27 g/L). The reference strain PO1f achieved this OD_600_ after 24 h (Fig. 1a), whereas the two modified strains (PO1f-CelB and PO1f-M1) needed 30 h (Fig. 1b, c). The OD_600_ of all three cultivations decreased slightly from 30 to 78 h and reached a final similar OD_600_ of ~ 40 (corresponding to dry biomass of ~ 20 g/L). During the cultivations, most of the cells stayed in the unicellular shape. Furthermore, the bioreactor cultivations showed that the integration of a single copy of the expression cassette at the AXP locus of PO1f-CelB and PO1f-M1 did not only led to a delayed growth of the cells but achieved the same maximum OD_600_.

Since both glycosidases showed β-galactosidase activity, an assay using *o*NPGal was used for the enzyme quantification. After 4 h of cultivation, the extracellular β-galactosidase activities were determined in the culture supernatants of PO1f-CelB and PO1f-M1 at 7.9 and 0.4 µkat_oNPGal_/L_medium_, respectively (Fig. 1b, c).

The highest volumetric *β*-galactosidase activities were measured at 187.5 µkat_oNPGal_/L_medium_ for CelB and 2.98 µkat_oNPGal_/L_medium_ for M1 during the stationary phase. The cultivation of the non-modified PO1f strain showed no endogenous *β*-galactosidase activity, as expected (Fig. 1a).

### Secretion efficiency of *Yarrowia lipolytica* for CelB and M1

**Fig. 2:**
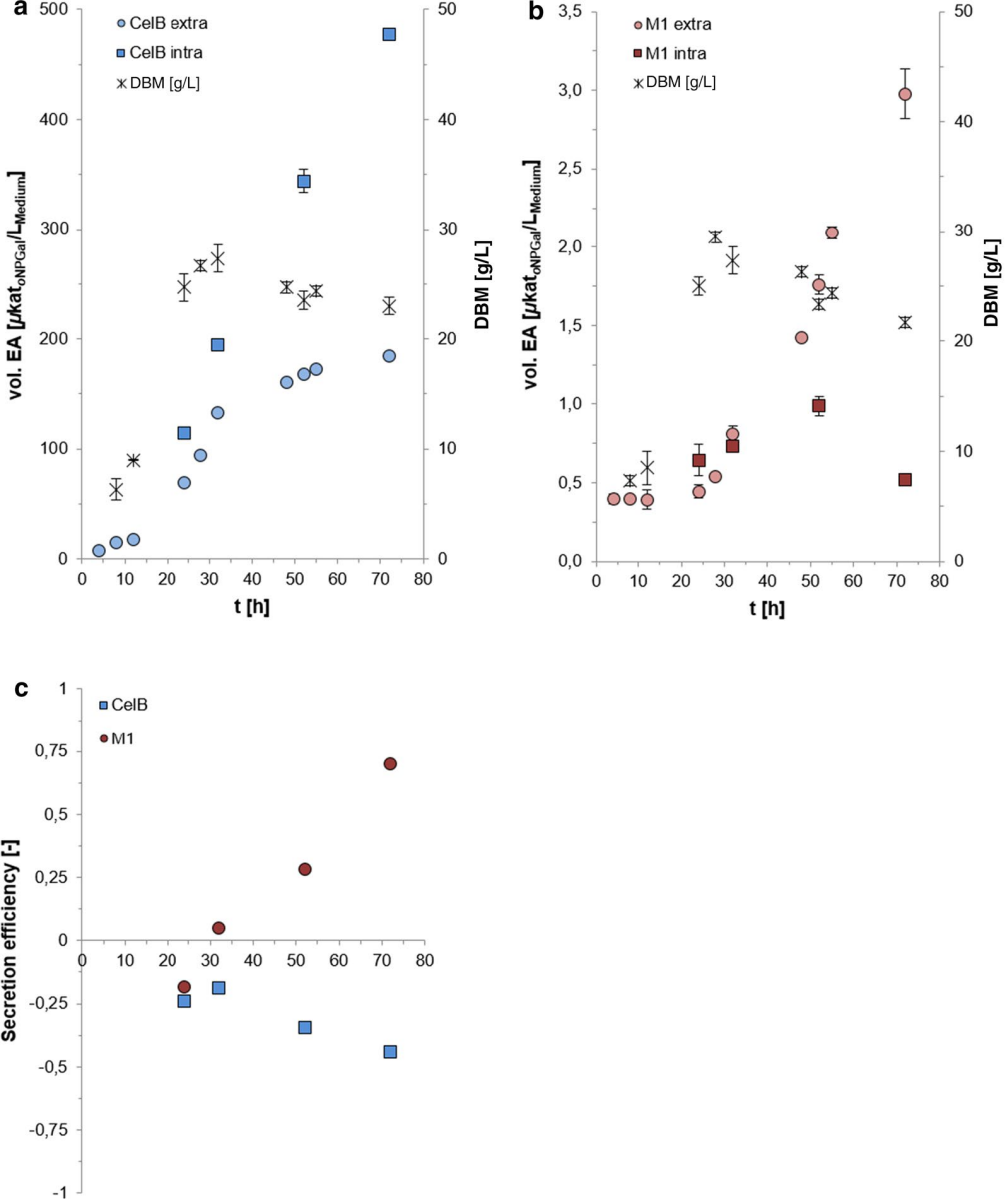
Comparison of intra- and extracellular β-galactosidase activities of CelB (**a**) and M1 (**b**). Secretion efficiency of *Y. lipolytica*-CelB and *Y. lipolytica*-M1 (**c**)

The intra- and extracellular β-galactosidase activity during the cultivation of both constructs, PO1f-CelB and PO1f-M1, was determined (Fig. 2). A significant difference between the amounts of intra- and extracellular β-galactosidase activities was observed. CelB was produced predominantly inside the cell (Fig. 2a), with nearly constant increasing amounts until the end of the cultivation. Only 28% of the total amount of CelB (184 µkat_oNPGal_/L_medium_) was found in the culture supernatant after 72 h. This suggests a secretion limitation of CelB while protein synthesis inside the cell proceeded. On the other hand, M1 was transported mainly outside the cell (Fig. 2b). After 72 h of cultivation, 85% of the total amount of M1 was secreted into the culture medium (2.98 µkat_oNPGal_/L_medium_). The secretion efficiency was defined as the difference between the extra- and intracellular β-galactosidase activity divided by the total β-galactosidase activity. A positive value (between 0 and 1) means more β-galactosidase activity was secreted outside the cell, while a negative value (between 0 and − 1) indicates the opposite. The result is shown in Fig. 2c. Surprisingly, the secretion efficiency of the large M1 (120 kDa) was positive and the smaller CelB (55 kDa) negative after 72 h (Fig. 2c). Although M1 was produced in much lower amounts (190-fold lower total activity) compared to CelB, the high molecular weight β-glycosidase showed clearly better secretion efficiency. It is suggested that the lower secretion efficiency of CelB contributes to an overload of the endoplasmatic reticulum and the initiation of the ER-associated protein degradation (ERAD) by the highly transcribed CelB. As it was shown before by Pfeffer et al. [35] where 60% of the newly synthesized recombinant antibody fragment Fab3H6 (47 kDa) was intracellularly degraded and not secreted by *Komagataella* sp.

### Correlation between the transcription of genes and corresponding β-galactosidase activities

**Fig. 3:**
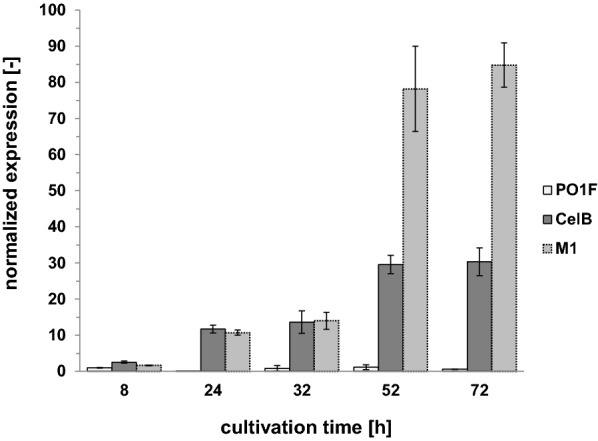
Quantification of recombinant gene expression in PO1f strains determined by qRT-PCR during bioreactor cultivation

**Fig. 4:**
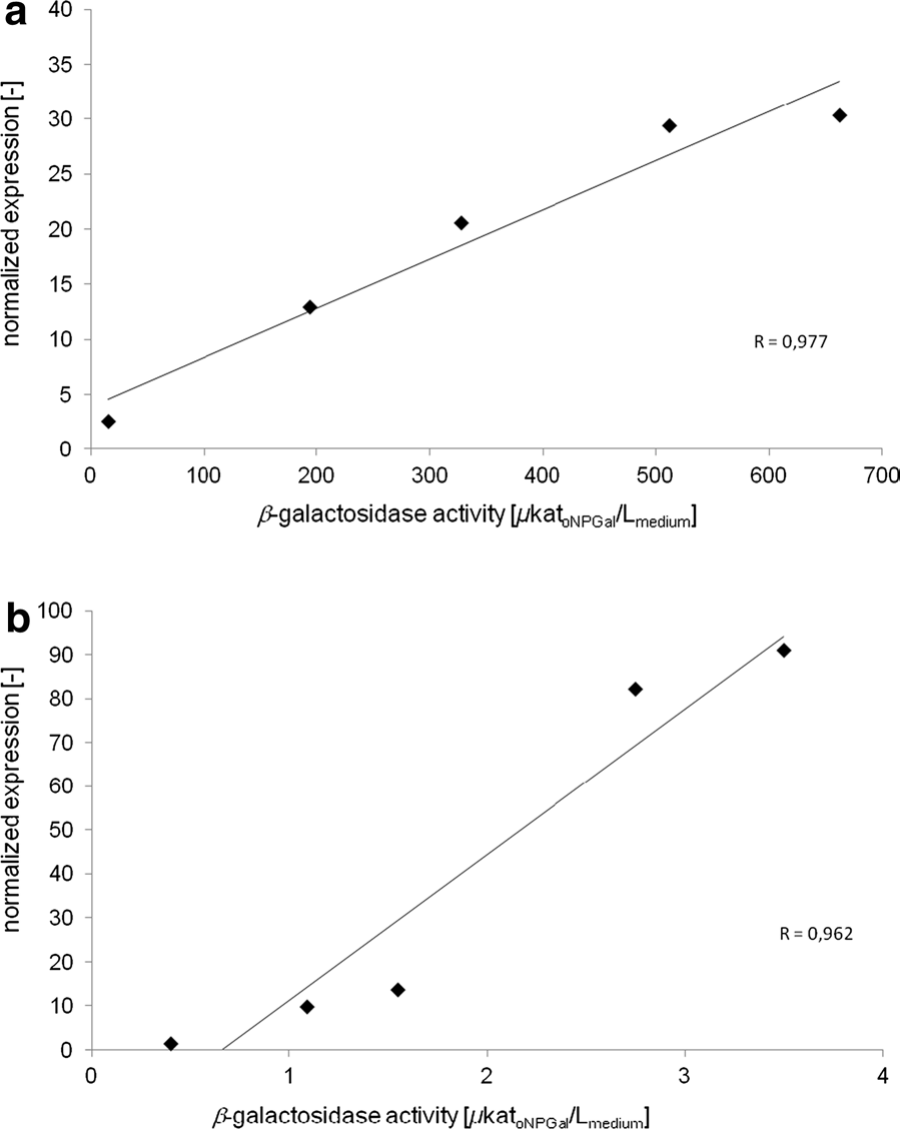
Correlation of transcript level and extracellular enzyme activity during bioreactor cultivation. Relative transcript level measured by qRT-PCR was plotted against total *β*-galactosidase activity of CelB (**a**) and M1 (**b**), respectively

The qPCR was used to determine the expression of the two recombinant genes *celB* and *M1* during the bioreactor cultivations. The primer pair of the genes of interest was targeted towards the signal sequence of the *XPR2* gene and, therefore, could be used for the detection of the mRNA amount of both recombinant genes. The unmodified *Y. lipolytica* PO1f strain (reference) was used as the calibrator and set to 1. All other values are shown as normalized expressions related to the reference using the ΔΔCT method for evaluation [36]. A first increase of the mRNA amount (about tenfold) for CelB and M1 was observed after 24 h (Fig. 3), which fits well to the total β-galactosidase activity increase of the CelB cultivation but not to the M1 production. During the next 8 h, the mRNA amount of CelB and M1 was almost the same. In the time frame from 8 to 32 h of cultivation, the total β-galactosidase activity of PO1f-CelB increased 22-fold from 14.8 to 327.5 µkat_oNPGal_/L_medium_. Although the mRNA amount of M1 increased similarly, the β-galactosidase activity here increased only fourfold (0.4 to 1.55 µkat_oNPGal_/L_medium_). After 52 h of cultivation, the second increase of the CelB and M1 mRNA amount was observed: For CelB, it was nearly doubled and for M1, it increased sixfold (Fig. 3). Again, a correlation to *β*-galactosidase activity increase was observed for PO1f-CelB (twofold from 327.5 to 662.3 µkat_oNPGal_/L_medium_). For PO1f-M1, the total β-galactosidase activity was just doubled (from 1.55 to 3.50 µkat_oNPGal_/L_medium_) but not sixfold higher, as indicated by the mRNA amount measured. Figure 4 shows the plot of total β-galactosidase activity against the amount of mRNA. A linear correlation was observed for CelB (Fig. 4a), while the M1 mRNA was less linearly correlated (Fig. 4b). However, the Pearson product correlation factor of CelB and M1 resulted in a strong positive correlation for both genes (rCelB = 0.977 and rM1 = 0.962). This study showed that although both genes were embedded in identical expression cassettes, the larger M1 gene was transcribed nearly three times more than the CelB gene. Contrarily, PO1f-M1 showed lower total β-galactosidase activities compared to PO1f-CelB, which probably indicated problems for M1 synthesis at the translational level (not investigated further).

### Partial secretome analysis of culture supernatants by SDS-PAGE and mass spectrometry

**Fig. 5:**
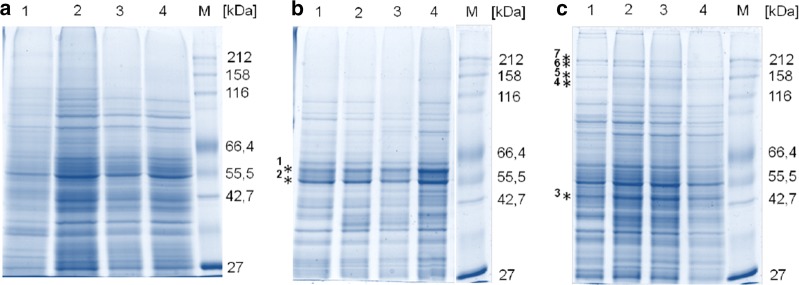
SDS-PAGE of *Y. lipolytica* PO1f (**a**), PO1f-CelB (**b**) and PO1f-M1 (**c**) secretome. Lanes 1–4 show samples taken after 24, 32, 52 and 72 h, respectively, from cultivation supernatants. The asterisks mark protein bands analyzed by mass spectrometry. A quantity of 5 µg protein was loaded for each lane

Samples from the culture supernatants were run on SDS-PAGE and characteristic bands were further analyzed by mass spectrometry (MS) for the analysis of secreted proteins during the PO1f cultivations and the identification of the two recombinant glycosidases CelB and M1. Figure 5a shows the high number of naturally secreted proteins from *Y. lipolytica* PO1f during the cultivation. The protein patterns of the secretome did not distinguish significantly when CelB or M1 was produced (Fig. 5b, c). In addition, no obvious differences were observed between samples taken from 24 to 72 h of cultivation time. An additional band between 55 and 66 kDa is clearly visible in the cultivation of PO1f-CelB (Fig. 5b, asterisk 1), which corresponded with the recombinant CelB, verified by MS analysis. Interestingly, the supernatant of the PO1f-M1 cultivation showed several high molecular weight bands above 116 kDa (Fig. 5c) compared to PO1f and PO1f-CelB. Four of them were analyzed by MS (Fig. 5c, asterisks 4–7) to investigate whether these high molecular weight protein bands could be multimers of M1. Indeed, the M1 protein sequence was identified in all four protein bands with an average coverage between 32% (highest band) and 40% (lowest band) and significant peptide counts of 33–39. Neither of the secreted recombinant glycosidases contained any peptide sequences belonging to the N-terminal signal sequence, suggesting a proper targeting and secretion of both glycosidases. An additional strong band was visible at ~ 45 kDa in the low molecular weight range of all M1 samples (Fig. 5c, asterisk 3) and, therefore, was subjected to MS analysis as well. The protein band contained mainly the sequence for the 3-phosphoglycerate kinase (AAC37504.1) with a size of 45 kDa and sequence coverage of 83%. Additionally, one of the most dominant protein bands in all three PO1f cultivations (Fig. 5b, asterisk 2) was identified as a so-called “hypothetical protein” (XP_505509.1) with a molecular weight of 47 kDa, which had a high similarity (> 73%) to the sequence of enolases (EC 4.2.11) from *Saccharomyces cerevisiae* (AAA88713.1) and *Candida albicans* (AAA71939.1). The amino acid sequence blasts from the sequences of four protein bands (Fig. 5; asterisk 1, 2, 3, 4) against the proteome of *Y. lipolytica* with the best hits are shown in Additional file 1: Figure S2.

Interestingly, neither highly abundant endogenous proteins in the M1 (enolase and 3-phosphoglycerate kinase) and CelB (enolase) culture supernatants has a N-terminal signal sequence on the gene level and, therefore, are predicted to be localized intracellularly. However, recent advances in secretome analysis showed that metabolic enzymes and heat shock proteins are secreted without recognized signal peptides by unconventional protein secretion (UPS) [37]. The enolase (EC 4.2.11) is described as being secreted using the UPS in several fungi and yeast species and was found in extracellular vesicles isolated from *Candida albicans* [38], *Cryptococcus neoformans* [39], *Paracoccidioides brasiliensis* [40] and *Saccharomyces cerevisiae* [41]. In addition, the 3-phosphoglycerate kinase was also detected as an extracellular protein in independent reports [42] and, therefore, is assumed to be possibly unconventionally secreted.

## Discussion

The two β-glycosidases, M1 and CelB, were expressed in the same genetic background by HR-mediated integration of both genes within the same expression cassette. Due to low HR-mediated integration efficiencies of foreign genes into the genome of *Yarrowia lipolytica* in general [43, 44], the CRISPR/Cas9 system was used to support the gene integration. Thereby, we achieved a HR efficiency up to 80%, as shown for the integration rate of the CelB expression cassette at the *AXP* locus. Schwartz et al. [33] had a lower integration rate of 62 ± 10% for the *hrGFP* gene embedded in the same expression cassette and at the same locus, although the *hrGFP* gene is shorter [33]. This showed an impact of the gene sequence, and not only the length, on the HR efficiency. Additionally, it was often observed that the HR efficiency decreases when larger genes were integrated in the same locus or if gene length was longer than the length of flanking regions [33, 43]. This phenomenon was also observed in our study, where the CelB expression cassette (80%; 2810 bp) was integrated significantly more efficiently than the larger M1 expression cassette (30%; 4511 bp).

The two recombinant PO1f-strains (PO1f-CelB and PO1f-M1) and the PO1f reference strain were investigated in bioreactor cultivations regarding growth behavior, gene expression, enzyme production and secretion. We observed a similar growth behavior in YPD complex medium for all three *Y. lipolytica* strains, where the culture reached the maximal OD after 32 h and then slightly decreased (Fig. 1), a course which was also recorded earlier by De Pourcq and coworkers [44].

*Yarrowia lipolytica* PO1f was able to produce and secrete both β-glycosidases when using the same constitutive promoter (UAS1B8_TEF(136)), N-terminal signal sequence and integration site for a single expression cassette under equal cultivation conditions. Surprisingly, while the smaller β-glycosidase (CelB with 55 kDa) was better expressed and showed much higher total enzyme activities than the larger M1 with 120 kDa, we only observed a positive secretion efficiency for the larger M1 (Fig. 2c). This showed that *Y. lipolytica* was able to secrete proteins up to a molecular weight of 120 kDa and that more factors besides size influence the secretion efficiency of a host. For example, the complexity of ternary and quaternary structure of proteins with its posttranslational modifications. So, both enzymes were checked for putative glycosylation sites. The amino acid sequences of both genes were analyzed. While the sequence of CelB did not harbor a consensus sequence for *N*-glycosylation, M1 contained eight of them. Therefore, we reinvestigated the mass spectrometry data of M1 with focus on diagnostic marker ions which are typical for *N*-glycosylation. None of the spectra from M1 did show these diagnostic marker ions, however glycosylation of M1 cannot be excluded, because not all putative *N*-glycosylation sites were detected by MS (Additional file 1: Figure S2).

The CelB *β*-glucosidase has been successfully expressed and secreted by *S. cerevisiae* [45] and *K. phaffii* [32] with 10 mg/L and 740 mg/L, respectively. However, it should be mentioned that the latter value (740 mg/L) corresponded to the total protein amount in the cultivation supernatant of *K. phaffii* and the authors did not analyze the intracellular CelB level. In our study, ~ 40 mg/L CelB was secreted by *Y.* *lipolytica* PO1f and, similar to the secretion study of *S. cerevisiae,* we observed high amounts of intracellular CelB. Only 28% of the CelB activity was found outside the cell after 72 h (Fig. 2a), whereas the secretion of CelB by *S. cerevisiae* was only ~ 15% and half of the intracellular CelB was not active [45]. We did not observe an accumulation of inactive CelB inside the cells, since the intracellular activity increased linearly and no strong CelB protein band was visible by SDS-PAGE of the intracellular fractions (Additional file 1: Figure S3). Most protein processing steps, such as the folding, disulfide formation [46] and the quality control, take place within the endoplasmic reticulum (ER) [47, 48]. The fact that we observed a continuous increase in intracellular CelB activity together with a linear increase in mRNA amount (Fig. 3) suggested a possible bottleneck during vesicle transport from the ER to the Golgi or from the Golgi to the outer membrane of *Y. lipolytica*. Since we observed an accumulation of active CelB inside the cell (Fig. 2a), investigation of alternative signal sequences might be promising to improve the secretion of CelB in *Y.* *lipolytica* in the future.

On the other hand, the secretion of the high molecular weight M1 was obviously not affected by bottlenecks within the secretory pathway. However, this could be caused, at least in part, by the low translation efficiency, which was indicated by the accumulation of mRNA after 52 h of cultivation (Fig. 3) and simultaneous low M1 activities (Fig. 2b). Secretion efficiency is tightly coupled with the translation efficiency within the co-translational pathway and vice versa. After the signal recognition particle (SRP) recognizes the early emerged polypeptide chain and binds to the signal peptide a stop or at least slow-down of translation elongation is induced [49]. Therefore, defaults in both, protein secretion and translational could have an impact on mRNA level. One possibility to circumvent this limitation in the future could be to alter the codon adaption of the M1 gene. Although we have already used a codon-optimized synthetic construct, it is often difficult to obtain a biased nucleotide sequence of a foreign gene when using computational optimization tools. Thorough evaluation of the synthetic gene sequence has uncovered some unbiased regions within the gene and will be investigated in further studies. Another possibility would be to test multiple promoters, because a strong promoter does not always optimize protein production and activity [50]. Since this was the first time that the M1 glycosidase was produced secretorily, there are no other studies for a direct comparison. In addition, this was the first time that an enzyme with a molecular weight of 120 kDa was being secreted by *Y. lipolytica*. As it has already been mentioned, the 90 kDa leucine amino peptidase II from *Aspergillus oryzae* [21] and glucoamylase from *Arxula adeninivorans* [20] were the proteins with the highest molecular weights secreted to date.

In addition to the two recombinant *β*-glycosidases, we observed several endogenous proteins which were secreted by *Y. lipolytica* PO1f (Fig. 5). Two proteins identified in the culture supernatant of *Y. lipolytica* PO1f, namely the 3-phosphoglycerate kinase and enolase (Fig. 5 and Additional file 1: Figure S2), did not carry a signal peptide. Nevertheless, some studies revealed that both enzymes were also localized extracellularly in other yeasts and fungi, secreted by UPS pathways [37–42, 51]. One general principle seems to be that most unconventional secretion events are non-constitutive and triggered by cellular stress [52]. It would probably be advantageous for the secretory production of recombinant enzymes with *Y.* *lipolytica* to avoid cultivation conditions which lead to starvation or other stress factors in order to reduce UPS in the culture supernatant. However, little is known about the UPS pathways in the different yeast and fungi and nothing about the UPS in *Y.* *lipolytica* has been described in the literature yet.

## Conclusion

The yeast *Y. lipolytica* PO1f secreted the high molecular weight β-glycosidase M1 in low amounts but effectively, and mRNA analysis pointed to an insufficient translation. On the other hand, the low-size β-glycosidase CelB was produced in sufficient amounts but showed low secretion efficiency. Due to the accumulation of active CelB inside the cell, we suggested that there are bottlenecks in the secretion pathway after correct translocation and folding in the ER. If this were true, the modification of the signal peptide would lead to an improved transport from the ER to the Golgi and, therefore, to an increase of CelB secretion. Regarding M1 production, an alteration of the codon optimization of the M1 gene might improve protein synthesis. This study showed a straightforward approach to reveal bottlenecks during protein secretion. Furthermore, it was shown for the first time that *Y. lipolytica* is a promising host for the secretory production of large and complex proteins above 100 kDa.

## Methods

### Chemicals, enzymes and kits

The chemicals used in this study were of analytical grade and purchased from Carl Roth GmbH (Karlsruhe, Germany), Sigma Aldrich (St. Louis, USA) and Fisher Scientific (Hampton, USA). All enzymes used for molecular biology and the Luna^®^ Universal One-Step RT-qPCR Kit were obtained from New England Biolabs GmbH (NEB; Germany). ExTaq™ Polymerase for screening genomic DNA was ordered from TaKaRa Bio Group.

### Strains and media

*Escherichia coli* XL1 was used for the plasmid propagation and cloning procedures and was grown in Luria–Bertani medium at 37 °C containing the respective antibiotic (50 µg/mL kanamycin or 100 µg/mL ampicillin).

*Yarrowia lipolytica* PO1f strain [53] was obtained from the International Centre for Microbial Resources, France, and was also used for the integration of two β-glycosidase genes (Additional file 1: Table S1). Selective media plates (6.7 g/L YNB (yeast nitrogen base, BD™ Difco™), 0.9 g/L CSM (Yeast Synthetic Drop-out Medium Supplements without uracil, leucine and tryptophan (Y1771) from Sigma Aldrich, 15 g/L agar and 20 g/L glucose) were used for the screening of positive transformants. Bioreactor cultivations were carried out in yeast extract–peptone–dextrose (YPD) medium (10 g/L yeast extract, 20 g/L bacto peptone and 20 g/L glucose).

### Plasmid construction for CRISPR-based integration of expression cassettes

Plasmids and primers used in this study are listed in Additional file 1: Tables S1 and S2. The genes coding for CelB from *Pyrococcus furiosus* and the metagenome β-galactosidase M1 [24] carrying an N-terminal sequence coding for a 22 amino acid residues compromising the signal sequence from the alkaline extracellular peptidase gene *XPR2* (Additional file 1: Figure S1) [23] were codon optimized for *Y. lipolytica* and synthesized by Invitrogen (Thermo Fisher Scientific).

The genes were cloned into a pHR_MFE1_hrGFP vector (derived from Ian Wheeldon, University of California [33]) by *NheI* and *BssHII* restriction to obtain vectors encoding for the expression cassette consisting of a UAS1B8_TEF(136) promoter [50], the respective gene of interest and a *CYC* terminator flanked by homologous regions for the MFE1 locus. The pHR vector backbone was amplified with the primers pHR_bb_fw and pHR_bb_rev using pHR_MFE1_hrGFP as template DNA to construct a pHR vector with 1 kb flanking regions for the acid extracellular peptidase (AXP) locus. Additionally, the two flanking regions of the AXP locus were amplified with primers pHR_FR1–FR4 from the genomic DNA of *Y. lipolytica* PO1f. The three fragments were assembled using NEBuilder^®^ HiFi DNA Assembly Master Mix (NEB, Germany) resulting in pHR_AXP. The expression cassette carrying either the *celB* or *M1* gene was cloned from pHR-MFE1 to pHR_AXP by *SpeI* and *AvrII* restriction, resulting in pHR_AXP_celB and pHR_AXP_M1, respectively.

Double strand breaks were induced by the CRISPR Cas9 system, which was encoded by the pCRIPSPRyl vector (derived from Ian Wheeldon, University of California [54]) for targeted integration of the expression cassette into the genome of PO1f. A 20 bp sgRNA was introduced into the vector to guide the Cas9 protein to the AXP region. The sgRNA sequence was designed using the CRISPOR website (http://crispor.tefor.net) and 20 base pairs, which were homologous to the integration site of the pCRISPRyl vector, were added up- und downstream (Additional file 1: Table S1 AXP_sgRNA) for integration of the sgRNA into the linearized and dephosphorylated pCRISPRyl vector. Linearization was carried out by digestion with 2.5 units *AvrII* for 1 h at 37 °C, followed by dephosphorylation with 1 unit Shrimp Alkaline Phosphatase (rSAP) for 2 h at 37 °C. Afterwards, the vector was analyzed by a 0.5% (w/v) agarose gel and extracted using the GeneJET Gel Extraction Kit (Thermo Scientific GmbH). An amount of 150 ng linearized vector DNA was mixed with fivefold excess of the single-stranded primer DNA, which was integrated into the vector using the NEBuilder^®^ HiFi DNA Assembly Master Mix (NEB, Germany). Correct sgRNA integration was analyzed by digestion with *Avr*II. Additionally, the vector DNA was sequenced using Primer Seq_sgRNA to confirm proper integration.

### Strain construction and screening

A amount of 500 ng of each plasmid (pHR_AXP_CelB or pHR_AXP_M1 and pCRISPRyl) was transformed into *Y. lipolytica* PO1f by electroporation for CRISPR-mediated integration of the expression cassettes into the AXP locus of PO1f [55]. Therefore, 50 mL of an overnight culture was pelleted by centrifugation at 8000*g* for 5 min and incubated in 8 mL of a solution containing 0.6 M sorbitol, 10 mM Tris HCl at a pH 7.5, and 150 mM lithium acetate for 1 h. After the cells has been washed twice with 1 M sorbitol, 10^9^ cells were electroporated by a single pulse at 2 kV for 4 ms (MicroPulser™; Bio Rad Laboratories). Subsequently, 900 µL 1 M sorbitol was added and the cells were plated onto selective agar plates (YNB + CSM) and grown for 2 days at 30 °C. Positive transformants were screened by genomic DNA PCR screening with Primers binding up- (AXP_screen_fw) and downstream (AXP_screen_rev) of the expression cassette, according to [54]. The genomic DNA was isolated, according to Lõoke et al. [56], by glass bead beating followed by phenol–chloroform extraction (method C). A quantity of 100 ng of the purified DNA was used as a template for the PCR screening. Candidates containing an integrated expression cassette were further cultivated overnight in YPD medium supplemented with 5-FOA (1 mg/mL) to remove the plasmids and were then plated onto YPD agar plates. The resulting recombinant PO1f strains were named PO1f-CelB and PO1f-M1, each with a single copy integration of the respective expression cassette in the AXP locus.

### Bioreactor cultivation of PO1f strains

Cultivation of *Y. lipolytica* PO1f candidates (PO1f, PO1f-CelB, PO1f-M1) was carried out in 450 mL YPD at pH 6.5 using the 1 L Multifors bioreactor system (Infors HT). The pH was kept constant at 6.5 with 2 M NaOH and 2 M H_3_PO_4_ solutions and the culture was aerated constantly with 0.5 vvm air (0.25 L/min), whereby the dissolved oxygen concentration pO_2_ was kept above 20% by increasing the stirrer speed stepwise. 405-DPAS-SC-K8S pH and InPro 6900 sensors of Mettler Toledo were used to measure the pH and pO_2_, respectively. Additionally, 100 µL of Antifoam 204 (Sigma, Germany) was added to each bioreactor to prevent foam formation. Cultures of each *Y. lipolytica* PO1f strain in YPD (50 mL) were cultivated at 30 °C in 500-mL shaking flasks for 20 h at 120 rpm up to an OD_600_ of 10. This culture was used to inoculate 450 mL YPD medium in the bioreactor. The PO1f strains were further cultivated for 78 h at 28 °C. Various 10-mL samples were taken after 4, 8, 12, 24, 28, 32, 48, 52, 55, 72 and 78 h to determine the intra- and extracellular β-galactosidase activity for the first cultivation. Samples were taken after 24, 32, 52 and 72 h for the second cultivation. Optical density (OD_600_) and dry biomass were monitored additionally every 2 h within the first 12 h, and later in the same interval, as described above. Two independent bioreactor cultivations for each strain were carried out and all measurements were taken in triplicate.

### Sample preparation and determination of protein concentration

The cells were separated from the medium by centrifugation (8000*g*, 5 min, 4 °C) for the determination of enzyme activity. A quantity of 2.5 mL of the supernatant was loaded onto a PD-10 column (GE Healthcare, Germany) and eluted with 3.5 mL sodium acetate buffer (50 mM, pH 5) for PO1f-CelB samples and 100 mM potassium phosphate buffer (100 mM, pH 6.75) with MgCl_2_ (5 mM) for PO1f-M1 samples. The corresponding cell pellet was resuspended in buffer to receive a 30% (w/v) cell suspension which was disrupted using a mechanical cell disruptor (OneShot, Constant Systems Limited, UK) applying a pressure of 1.5 kbar. After disruption, the cell debris was separated from the intracellular soluble proteins by centrifugation (13,000*g*, 15 min, 4 °C). Buffered samples were further used for the determination of the protein concentration, according to the method of Bradford [57], using bovine serum albumin as a standard.

### Determination of intra- and extracellular β-galactosidase activity

The enzyme activity of CelB and M1 was determined as described previously [24], using *o*NPGal (*o*-nitrophenyl-β-d-galactopyranoside) as the substrate. Therefore, 100 µL of samples were added to a mixture of 500 µL buffer (50 mM sodium acetate buffer pH 5 for CelB and 100 mM potassium phosphate buffer pH 6.75 with 5 mM MgCl_2_ for M1) and 600 µL 50 mM *o*NPGal dissolved in the respective buffer. Reaction took place at 75 and 37 °C for CelB and M1 samples, respectively, and the absorption change at 405 nm was measured for 2 min in a temperature-controlled cuvette (Ultrospec 3000, Amersham Bioscience, Germany). All components were preincubated for 5 min at the corresponding temperature. One katal was defined as the amount of enzyme that catalyzes the release of 1 mol *o*-nitrophenol from *o*NPGal per s. The enzyme activity of each sample was determined in triplicate.

### SDS-PAGE analysis of *Y. lipolytica* secretome

The protein pattern of the extracellular secretome of each strain was visualized by sodium dodecyl sulfate (SDS) polyacrylamide gel electrophoresis (PAGE) using a 10% separation gel [58]. An amount of 10 µg protein was precipitated with TCA (20% (v/v)), incubated overnight at 4 °C, washed with acetone and resuspended in SDS sample buffer [0.02% (w/v) Tris–HCl, 6% (w/v) Glycerol, 0.1% (w/v) bromophenol blue, 4% (w/v) SDS and 2% (w/v) β-mercaptoethanol] for all samples analyzed. Samples were boiled for 5 min at 95 °C prior to loading onto the gel. The protein molecular weight marker (broad range 2–212 kDa, NEB) was loaded for reference. Proteins were visualized with Coomassie Brilliant Blue G-250 staining [59].

### In-gel digestion

Proteins were in-gel digested using trypsin (Roche, Germany), according to Shevchenko et al. [60]. After digestion, the supernatant was collected in a new tube, dried in a vacuum centrifuge and stored at − 20 °C. Dried samples were dissolved in 0.1% TFA for nanoLC-MS/MS analysis.

### NanoLC-MS/MS analysis

NanoLC-ESI–MS/MS experiments were carried out on an EASY-nLC 1000 system (Thermo Fisher Scientific, Germany) coupled to a Q-Exactive Plus mass spectrometer (Thermo Fisher Scientific, Germany) using an EASY-Spray nanoelectrospray ion source (Thermo Fisher Scientific, Germany). Tryptic peptides were injected directly into an EASY-Spray analytical column (2 μm, 100 Å PepMapRSLC C18, 25 cm × 75 μm, Thermo Fisher Scientific) operated at a constant temperature of 35 °C. Peptides were separated at a flow rate of 250 nL/min using a 30 min gradient with the following profile: 2–45% solvent B in 30 min, 45–70% solvent B in 10 min, 45–95% solvent B in 10 min, 15 min isocratic at 95% solvent B, followed by 95–2% solvent B in 5 min and re-equilibration at 2% solvent B for 15 min. The solvents used were 0.5% acetic acid (solvent A) and 0.5% acetic acid in ACN/H_2_O (80/20, v/v, solvent B). The Q-Exactive Plus was operated under the control of XCalibur 4.0 software. The MS spectra (m/z = 300–1600) were detected in the Orbitrap at a resolution of 70,000 (m/z = 200) using a maximum injection time of 100 ms and an automatic gain control (AGC) value of 1 × 10^6^. Internal calibration of the Orbitrap analyzer was carried out using lock-mass ions from ambient air, as described in Olsen et al. [61]. Data-dependent MS/MS spectra were generated for the 12 most abundant peptide precursors in the Orbitrap, using high energy collision dissociation fragmentation at a resolution of 35,000, normalized collision energy of 27 and intensity threshold of 1 × 10^5^. Only ions with charge states from + 2 to + 5 were selected for fragmentation, using an isolation width of 1.6 Da. The AGC was set at 5 × 10^5^ and the maximum injection time was 100 ms for each MS/MS scan. Fragmented precursor ions were dynamically excluded for 30 s within a 5-ppm mass window to avoid repeated fragmentation.

### MS data analysis

Mascot 2.6 (Matrix Science, UK) was used as a search engine for protein identification. Spectra were searched against a *Yarrowia lipolytica* protein sequence database downloaded as FASTA-formatted sequences from UniProt [62] and the two reference sequences of the β-glycosidases. Search parameters specified trypsin as the cleaving enzyme, a 5-ppm mass tolerance for peptide precursors and 0.02 Da for fragment ions. Carbamidomethylation of cysteine residues was defined as a fixed modification. Methionine oxidation was allowed as a variable modification. Mascot search results were imported into Scaffold version 4.8.6. (Proteome Software, USA). Peptide identifications were accepted with a peptide probability greater than 80.0%, as specified by the Peptide Prophet algorithm [63]. Proteins had to be identified by at least two peptides and a protein probability of at least 99% to be accepted. Protein probabilities were assigned by the Protein Prophet algorithm [64].

### Quantitative reverse transcription PCR

The total RNA of the different *Y. lipolytica* PO1f strains was extracted from 5 × 10^8^ cells after 8, 24, 32, 52 and 72 h using the innuSPEED Bacterial/Fungi RNA Kit (Analytik Jena). The quantitative reverse transcription PCR (qRT-PCR) was carried out in a one-step reaction using the Luna^®^ Universal One-Step RT-qPCR kit. A total RNA amount of 7 ng was applied for combined cDNA synthesis and quantitative real/time PCR (qRT-PCR) using the primer pair which was targeted against the signal sequence of the alkaline extracellular protease (RT_SP_fw and RT_SP_rev, Additional file 1: Table S2). The primers amplify a 66 bp-long sequence of the signal sequence at the 5′-end of the recombinant *celB* and *M1* genes, respectively. The actin gene of *Y. lipolytica* was used as a reference gene (Additional file 1: Table S2; RT_actin_fw and RT_actin_rev). qRT-PCR was carried out in triplicate in a 96-well plate using an Analytik Jena pTOWER2.2 device. Cycle threshold (Ct) values could be measured for the gene of interest (*celB* and *M1*) and the reference gene (actin), respectively, at an annealing temperature of 59 °C. A reaction protocol was used: 55 °C for 10 min, 95 °C for 1 min, 40 cycles at 95 °C for 10 s and 59 °C for 30 s. We observed specific melting temperatures at 84 and 85 °C for the gene of interest and reference product, respectively. The relative mRNA quantity of the two recombinant genes was determined by the ΔΔCT method, resulting in a relative expression level in comparison to a calibrator. The sample taken from *Y. lipolytica* PO1f (without an integrated expression cassette) after 8 h of cultivation was used as the calibrator and, therefore, was set to 1. All other transcription rates were given in relation to this value as normalized expression levels.

### Statistical analysis

Standard deviation was used for data evaluation and calculated with Excel. All experiments were conducted at least in duplicate, with three independent measurements.

## Supplementary information

**Additional file 1: Figure S1.** Used *XPR2* signal peptide, consisting of a pre-peptide and a dipeptidyl stretch (DPS). The amino acid and DNA sequence is shown. **Figure S2.** Amino acid sequence blast of four protein bands (Fig. 5b asterisk 1, b2, c3 and c4) analyzed by mass spectrometry with highest coverage and unique peptide counts. For better understanding the gene name of 3-phosphoglycerate kinase was introduced into figure file afterwards. Filled black triangles indicate the cleavage site of the signal peptide. Putative N-glycosylation sites are underlined. **Figure S3**. SDS-PAGE of *Y. lipolytica* PO1f (A), PO1f-CelB (B) and PO1f-M1 (C) intracellular protein pattern. Lanes 1–4 show samples taken after 24, 32, 52 and 72 h, after cell disruption. A quantity of 5 µg protein was loaded for each lane. **Table S1.** Strain and plasmids used in this study. **Table S2.** Primers used in this study.

## Data Availability

All data generated or analyzed during this study are included in this published article [and its additional information files].
