# Supplementary material for: Secretion of a low and high molecular weight β-glycosidase by Yarrowia lipolytica
Source: Microb Cell Fact. 2020 May 11;19:100. doi: 10.1186/s12934-020-01358-5 (PMC7216700; doi:10.1186/s12934-020-01358-5)
Supplement: Supplementary file 1 — Additional file 1: Figure S1. Used XPR2 signal peptide, consisting of a pre-peptide and a dipeptidyl stretch (DPS). The amino acid and DNA sequence is shown. Figure S2. Amino acid sequence blast of four protein bands (Fig. 5b asterisk 1, b2, c3 and c4) analyzed by mass spectrometry with highest coverage and unique peptide counts. For better understanding the gene name of 3-phosphoglycerate kinase was introduced into figure file afterwards. Filled black triangles indicate the cleavage site of the signal peptide. Putative N-glycosylation sites are underlined. Figure S3. SDS-PAGE of Y. lipolytica PO1f (A), PO1f-CelB (B) and PO1f-M1 (C) intracellular protein pattern. Lanes 1–4 show samples taken after 24, 32, 52 and 72 h, after cell disruption. A quantity of 5 µg protein was loaded for each lane. Table S1. Strain and plasmids used in this study. Table S2. Primers used in this study. [file 12934_2020_1358_MOESM1_ESM.pdf]

Additional file 1

Secretion of a low and high molecular weight β-glycosidase by *Yarrowia lipolytica*

Paul Swietalski, Frank Hetzel, Ines Seitl, Lutz Fischer*

University of Hohenheim, Institute of Food Science and Biotechnology,

Department of Biotechnology and Enzyme Science,

Garbenstr. 25, 70599 Stuttgart, Germany

*Corresponding author:

E-mail address: lutz.fischer@uni-hohenheim.de

Tel.: +49 711 459 23588

Keywords: *Yarrowia lipolytica*, PO1f, CRISPR Cas9, Protein secretion, Recombinant enzyme production, Yeast expression system, Unconventional protein secretion, CelB, M1

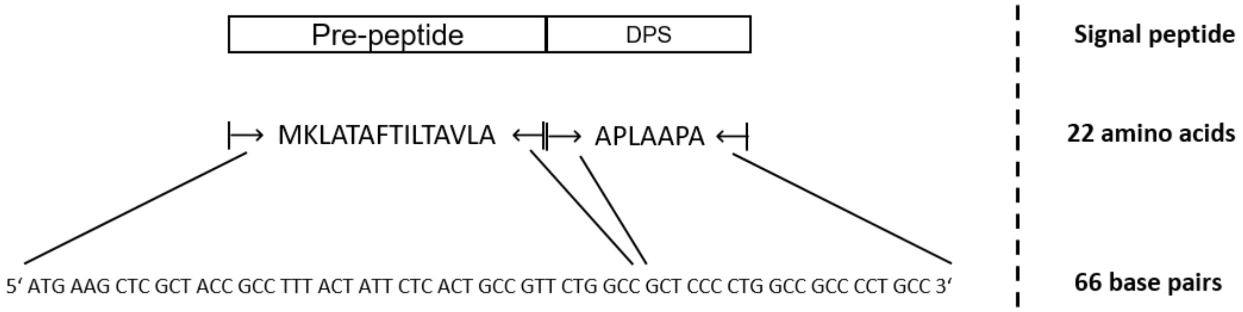

Additional Figure 1.

A NP_000000.564 (100%), 120.958,5 Da
AKO22784_1_M1
39 exclusive unique peptides, 63 exclusive unique spectra, 113 total spectra, 403/1060 amino acids (38% coverage)

```
MKLATAFTIL TAVLAAPLAA PARQKLVYIP PANGYPEWDN NPELFQVNRM
EAHATWIPFD TKEEAFIGDF KASSNYLSLN GLWKFAYADT PDKRIRSFYE
QDYDCSSWAE LTVPSHWQLH GYDYPQYTNV NYPWSEREPE LKPPFAPSKY
NPVGSYVRTF TVPEGWVEKP VFISFQGVES AFYVWVNGDL VGYSEDTFTP
AEFDITPYLT PGENKLAVEV YKWCDASWLE DQDFWRLSGI FRDVYLYTAP
ETHIYDFFVR TELDDQYRHA ELQLDVKLKD YFARTTEAIH VDAELYDHNQ
DPVFDNPLSQ IINFNGASNQ TFSLSASVLD PLKWSAEQPN LYTLILSLRN
DNGDLLEVVR CRVGFRKFEL KDGLMKINGQ RIVFKGVNRH EFSCDTGRAI
SVDDMIQDIQ LMKAHNINAV RTSHYPNQTT WYELCDEYGL YVIDETNLET
HGSWSYGQKE LGEHTVPGSK PEWRDNVLDR CNSMFQRDKN HPSIIIWSLG
NESFGGDNFI SMYDFLKQED PTRLVHYEGQ FHYRESSAAS DIESTMYVKP
VDVEKYALND PKKPYIICEY SHAMGNSCGG LHLYWELFEK YDILQGAFIW
DWIDQAIRIE QADGTVQMAY GGDFGESPHD GNFCGNGLIF ADHIVSPKLY
EVKKCYQNVK FEAVDIDSGH IRVTNRNLFT DLAEYDLKWE VACNGQIVQT
GGIDLAVPAG DVAEITVPFV HVNESTPDGE YVLTLSMVLK EATLWAEVGH
ELAWEQFVLP NSRHEALPTQ TTSSDSVLVK EQADQLIVQV ADVSLQFNKS
NGCLSSIQNA GEELLLEPVR PNFWRAVTDN DLGNKLNERC AVWRTVWDTS
TLNSFEWHKD SDDVIVRAEY SLHTEPVSSL TLEYKVGSNG SINVFEEFSP
GKGLPEIPEI GMLFVLKDSL NTVSWYGRGP HENYWDRKTG ARLGYFSGRI
QDQFVPYIRP QECGNKTDVR YASITQGTNG SGIHIEGDPV LELNALPWTP
EELEAHDHVY KLPASNKTVV RVNYKQMGVG GDDSWNAPTH EEFTLPADKT
YGFDFTFRTI
```

B NP_000000.512 (100%), 56.673,5 Da
Cel_B
43 exclusive unique peptides, 78 exclusive unique spectra, 176 total spectra, 352/493 amino acids (71% coverage)

```
MKLATAFTIL TAVLAAPLAA PAKFPKNFMF GYSWSGFQFE MGLPGSEVES
DWWVWVHDKE NIASGLVSGD LPENGPAYWH LYKQDHDIAE KLGMDCIRGG
IEWARIFPKP TFDVKVDVEK DEEGNIISVD VPESTIKELE KIANMEALEH
YRKIYSDWKE RGKTFILNLY HWPLPLWIHD PIAVRKLGPD RAPAGWLDEK
TVVEFVKFAA FVAYHLDDLV DMWSTMNEPN VVYNQGYINL RSGFPPGYLS
FEAAEKAKFN LIQAHIGAYD AIKEYSEKSV GVIYAFAWHD PLAEEYKDEV
EEIRKKDYEF VTILHSKGKL DWIGVNYYSR LVYGAKDGHL VPLPGYGFMS
ERGGFAKSGR PASDFGWEMY PEGLENLLKY LNNAYELPMI ITENGMADAA
DRYRPHYLVS HLKAVYNAMK EGADVRGYLH WSLTDNYEWA QGFRMRFGLV
YVDFETKKRY LRPSALVFRE IATQKEIPEE LAHLADLKFV TRK
```

C XP_505509.1 (100%), 47.307,7 Da
YALI0F16819p [Yarrowia lipolytica CLIB122]
37 exclusive unique peptides, 62 exclusive unique spectra, 234 total spectra, 282/438 amino acids (64% coverage)

```
MPVEKLHARY VYDSRGNPTV EVDLTTQHGL FRAIVPSGAS TGVHEALELR
DKDASKWGGK GVLKAVQNVN EIIAPAVIDA KLDVKDQAAF DKFLLELDGT
ENKSKLGANA ILGVSIAAAR AGAGEKGVPL YEHIASLASS PQPYVLPVPF
LNVLNGGSHA GGRLAIQEFM IAPTEFESFS ESLRAGTEVY HELKKLAKKE
YGASAGNVGD EGGVAPDIQT AEEALNLITE AIDAAGYTGK IKIAIDAASS
EFYNEEANKY DLDFKNPDSD KSLWKTGEEL AAYYSELIKK YPIVSFEDPF
AEDDWAAWSH FVSTTDIQIV GDDLTVTNPV RIKRAIEEKS ANALLLKVNQ
IGTITESIQA ANDSYDAKWG VMVSHRSGET EDVTIADLAV GLRAGQIKTG
APSRSERLAK YNQLLRIEEE LGDKAIFAGP KFHLSRAI
```

D AAC37504.1 (100%), 44.773,7 Da
3-phosphoglycerate kinase [Yarrowia lipolytica] , YALI0D12400g
40 exclusive unique peptides, 66 exclusive unique spectra, 95 total spectra, 345/417 amino acids (83% coverage)

```
MSLTNKLSIK DLDLKNKRVF IRVDFNVPLD GTTITNNQRI VAALPSIKYA
IDQGAKAVIL ASHLGRPNGQ RVEKYSLKPV QAELSKLLGK PVTFLDDCVG
PKVEEEVSKA KDGEVILLEN LRFHPEEEGS HKDADGKKIK ADPAKVEEFR
KSLTSLADVY VNDAFGTAHR AHSSMVGIEL PQRAAGFLVA KELEFFAKAL
EHPERPFLAI LGGAKVSDKI QLIDNLLDKV NALIIGGGMA FTFKKTLENV
KIGNSLFDED GSKIVAGLVE KAKKNNVKLV FPVDYVTADK FSKDAKVGHA
TDAEGIPDGW QGLDCGPKSI EEFQKVIGES KTILWNGPPG VFEFDNFAKG
TKAVLDACVK AVDNGATVII GGGDTATVAK KYGAEDKLSH VSTGGGASLE
LLEGKTLPGV AALSEKK
```

Additional Figure 2.

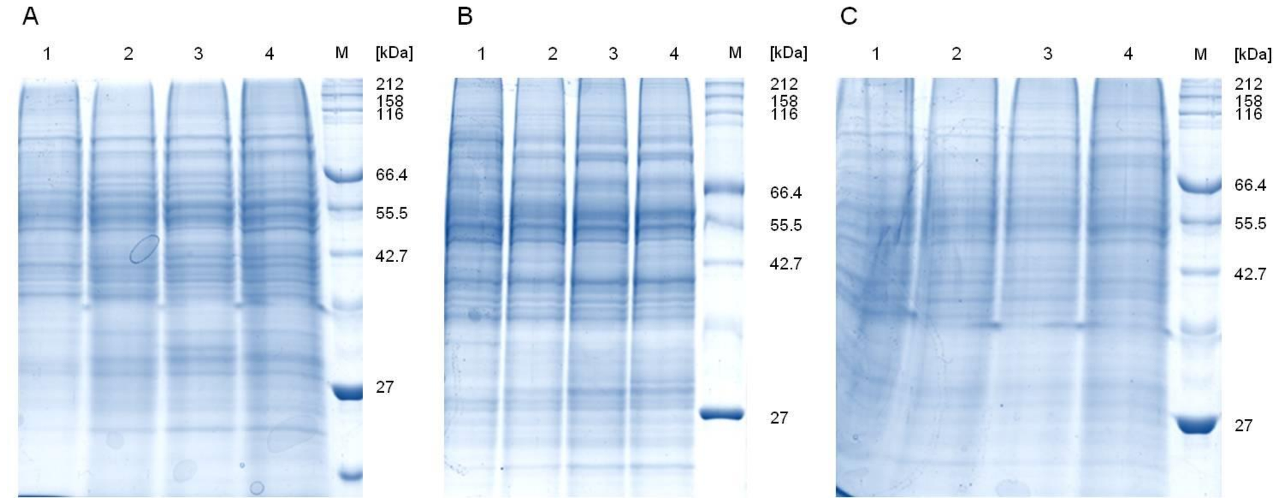

Additional Figure 3.

Additional Table 1.

	Characteristics	References
***Y. lipolytica* strains**		
PO1F	MatA, leu2-270, ura3-302, xpr2-322, axp-2 Leu-, Ura-, Suc+	CLIB 724
PO1F-CelB	MatA, leu2-270, ura3-302, xpr2-322, axp::celB Leu-, Ura-, Suc+	This work
PO1F-M1	MatA, leu2-270, ura3-302, xpr2-322, axp::M1 Leu-, Ura-, Suc+	This work
**Plasmids**		
pMK_preCelB_YLop	pMK-RQ (kanR; invitrogen) with codon optimized *celB* and N-terminal signal peptide	This work
pMK_preM1_YLop	pMK-RQ (kanR; invitrogen) with codon optimized *M1* and N-terminal signal peptide	This work
pCRISPRyl	Codon optimized Cas9 endonuclease and sgRNA integration site; Leu2 marker	(34)
pHR_MFE1_GFP	MFE1 sites for homologous recombination; expression cassette with hrGFP; Ura3 marker	(32)
pHR_MFE1_CelB	MFE1 sites for homologous recombination; expression cassette with preCelB_YLop; Ura3 marker	This work
pHR_AXP	AXP sites for homologous recombination; without expression cassette; Ura3 marker	This work
pHR_AXP_CelB	AXP sites for homologous recombination; expression cassette with preCelB_YLop; Ura3 marker	This work
pHR_AXP_M1	AXP sites for homologous recombination; expression cassette with preM1_YLop; Ura3 marker	This work

Additional Table 2.

Primer	Sequence [5´ - 3´]
AXP_sgRNA	GGGTCGGCGCAGGTTGACGTGACCAGGTCGAAGTAGTGGGTTTTAGAGCTAGAAATAGC
Seq_sgRNA	GTGTGAAAAGTTGGAGTG
AXP_FR1_up	GTTCGAAGGTACCAAGGAAGCATGCGGTACGAACGGCACCAGCTGG
AXP_FR2_up	CCTAGGGATCGTTGTCAGAAGTAAGACTAGTACCTGGTCCTCAGTCATTGC
AXP_FR3_down	ACTAGTCTTACTTCTGACAACGATCCCTAGGAAAAGTGGTTGTACCAGAAAACAG
AXP_FR4_down	AACAGCTATGACCATGATTACGCCAAGCTTAATTGCATCCAACAATGTTGAAC
pHR_bb_fw	GTACCGCATGCTTCCTTGG
pHR_bb_rev	AGCTTGGCGTAATCATGGTC
AXP_screen_fw	CTAAAGATGTTGATCTCCTTGTGCC
AXP_screen_rev	CCTCTGGGCCGAATACAACAC
RT_SP_fw	ATGAAGCTCGCTACCGCCTTTAC
RT_SP_rev	GGCAGGGGCGGCCAG
RT_actin_fw	TCCAGGCCGTCCTCTCCC
RT_actin_rev	GGCCAGCCATATCGAGTCGCA
